# An exploratory assessment of the preference for eHealth interventions to prevent HIV and sexually transmitted infections among men who have sex with men in Hanoi, Vietnam

**DOI:** 10.1186/s12889-020-09449-z

**Published:** 2020-09-11

**Authors:** Long Hoang Nguyen, Huong Lan Thi Nguyen, Mattias Larsson, Bach Xuan Tran, Mart L. Stein, Luis E. C. Rocha, Susanne Strömdahl

**Affiliations:** 1 Department of Global Public Health Sciences, Karolinska Institutet, SE-171 77 Stockholm, Sweden; 2 Center of Excellence in Behavioral Medicine, Nguyen Tat Thanh University, Ho Chi Minh City, Vietnam; 3 Institute for Global Health Innovations, Duy Tan University, Da Nang, Vietnam; 4 Department of Health Economics, Institute for Preventive Medicine and Public Health, Hanoi Medical University, Hanoi, Vietnam; 5 Department of Health, Behaviours, and Society, Johns Hopkins Bloomberg School of Public Health, Baltimore, MD USA; 6 National Coordination Centre for Communicable Disease Control, Centre for Infectious Disease Control, National Institute for Public Health and the Environment, Bilthoven, The Netherlands; 7 Department of Economics & Department of Physics and Astronomy, Ghent University, Ghent, Belgium; 8 Department of Medical Sciences, Section of Infectious Diseases, Uppsala University, Uppsala, Sweden

**Keywords:** Qualitative, Focus group, Men who have sex with men, Ehealth, Online intervention, Vietnam

## Abstract

**Background:**

Electronic health (eHealth) interventions are promising in HIV and sexually transmitted infections (STIs) prevention among men who have sex with men (MSM), given a high rate of the Internet use in this population. This study determined the preferences for eHealth interventions to prevent HIV and STIs among MSM in Hanoi, Vietnam to guide the development of future eHealth interventions.

**Methods:**

Five focus group discussions (FGD) were conducted with 35 MSM recruited by purposive sampling in January 2018 in Hanoi, Vietnam. The FGDs addressed attitudes towards the feasibility and uptake of HIV/STI interventions via online modalities such as smartphone applications (apps, social network sites, or emails); preferences and concerns regarding an online HIV/STI intervention. FGDs were audio-recorded and transcribed verbatim. Content analysis was used to determine themes.

**Results:**

MSM reported that they commonly searched for information regarding HIV/STI and sexual health on Facebook and a variety of mobile apps. They perceived a lack of reliable online sources, a high need, and interest for an online intervention. Most of them preferred short and concise messages without perceived sensitive words such as “HIV” or “STI”. Diversity of online modalities were preferred with information from credible sources about HIV/STI symptoms, testing and treatment, safe sex practices and testing locations with a focus on safe MSM-friendly clinics. Concerns about the need to trust the organization behind the online information and interventions, and the importance of confidentiality when participating in online interventions were raised.

**Conclusion:**

High acceptance and perceived need for an online HIV/STI intervention were reported. The importance of establishing trust within the MSM community as a reliable source of information was emphasized, as well as the importance of confidentiality.

## Background

Despite substantial progress in HIV prevention and treatment globally, men who have sex with men (MSM) continuously face an excessive burden of HIV and sexually transmitted infections (STIs) [1–3]. According to the 2017 UNAIDS report, approximately 12% of global new HIV infections in 2015 were attributed to MSM, which was significantly higher than in other most-at-risk populations such as female sex workers (5%) and people who inject drugs (8%) [4]. Causes of HIV transmission among MSM have been well documented, such as sexual risk behaviors including unprotected sexual intercourses and multiple sex partners, as well as a high frequency of substance use and abuse [2, 5–10]. However, many MSM living with HIV do not acknowledge their HIV serostatus, ranging from 16% in the United States [11], 21% in New Zealand [12], 22% in Spain [13] to 86% in China [14] and 90.4% in Malawi [15]. Consequently, they receive delayed care, which threatens their health and accelerates HIV progression [16, 17]. Therefore, interventions to promote behavioral changes and HIV/STIs testing uptake in this population should be highly considered when planning HIV prevention strategies.

In Vietnam, MSM are identified as one of the most-at-risk and stigmatized population for the national HIV epidemic [18]. In spite of efforts in offering prevention and treatment, the prevalence of HIV in Vietnamese MSM has been consistently increasing. The National HIV Sentinel Surveillance Survey (HSS) revealed that the overall HIV infection rate in the MSM population elevated from 5.2% in 2015 to 7.4% in 2016 [19]. However, the rates of sexual risk behaviors in this population are significantly high. Data from HIV Sentinel Surveillance Survey Plus in 2016 reported that approximately two-third of MSM (61.1%) did not utilize condoms with a male partner at the last anal intercourse [20]. Another survey indicated that 43.7% MSM in Hanoi and 70.4% MSM in Ho Chi Minh city had more than two concurrent male sex partners [21], and 30.4% MSM had ever used amphetamine -type-stimulants [22]. Nonetheless, the rate of HIV/STI testing services utilization in MSM in Vietnam was low across studies (19.2%–23.5%) [23, 24]. Various face-to-face behavioral interventions have been developed among MSM to diminish the burden of HIV and other sexually transmitted infections (STIs) [25–28]. However, these interventions faced challenges in maintaining their sustainability such as scarcity of resources, the stigma around homosexuality practices, limited MSM-advocacy and community organizations and insufficient MSM-oriented health professionals [29–31], requiring innovative solutions to address these issues.

The rapid expansion of Internet and mobile technology access raises a new approach so-called electronic health (eHealth) interventions, which can address the weaknesses of traditional interventions in HIV prevention, treatment, and care [25, 32–35]. For example, it can facilitate interventions’ accessibility given that MSM can access the intervention from almost anywhere over the Internet, at any time convenient, with a high sense of confidentiality, which is particularly important for MSM in places where homosexuality are stigmatized and/or criminalized [36, 37]. Moreover, MSM can easily connect with their community, MSM-friendly organizations or qualified health professionals by distance via chatrooms, social network applications or text messages [38, 39]. Evidence in worldwide has shown positive effects of eHealth interventions on promoting HIV/STI knowledge, increasing rates of condom use and HIV/STIs testing, as well as enhancing antiretroviral treatment adherence [40–42], which are comparable to the face-to-face approach [25]. A systematic review from 2014 revealed that eHealth interventions could diminish sexual risk behaviors for HIV/STIs and promote uptake of testing among MSM [37].

MSM in Vietnam have a high level of Internet and mobile devices access. Two national online surveys from 2008 to 2011 showed that 67–94% of MSM used the Internet regularly [43, 44]. Another study in Ho Chi Minh city revealed that 73.4% of MSM sought male sex partners via the Internet and 76.1% of MSM accepted to find HIV/AIDS-related information on the Internet [45]. The most recent evidence from 2016 indicated that 91.7% and 67.8% of MSM respectively had a smartphone and a computer/tablet, of whom 50.7% and 75.1% of MSM used mobile devices to find HIV/STIs testing clinics and HIV/STIs information, correspondingly [46]. The high rates of Internet use and mobile phone ownership in Vietnamese MSM offer a great potential to deliver HIV/STIs-related eHealth interventions to this most-at-risk population.

To date, eHealth interventions to support HIV prevention among MSM in Vietnam have been not available yet. The optimal design of eHealth intervention requires an in-depth investigation of the preference of the target group for the interventions’ content, presentation and visualization method. Previous reviews indicated the advantages and disadvantages of different eHealth interventions on MSM, which highly depended on study settings [37, 39]. In addition, understanding the patterns of electronic devices use, desired contents or functionality, as well as the unmet needs of the target population, is vital to develop successful interventions [47]. Thus, we conducted a qualitative study to determine the preferences for eHealth interventions to prevent HIV and STIs among MSM in Hanoi, Vietnam.

## Methods

### Study population and sampling method

The qualitative data collection was conducted in January 2018 in Hanoi, the capital of Vietnam. Hanoi is one of the settings in the country with the highest number of MSM alongside Ho Chi Minh city [44]. There is a global trend that MSM prefer to live in urban areas. A previous online survey in 2010 estimated that more than 30% of Vietnamese MSM lived in Hanoi [44]. The inclusion criteria for the focus group discussions (FGDs) were: i) male; ii) 18 years and above; iii) had sexual contact with at least one other man during the last 12 months, and iv) lived in Vietnam during the last 12 months.

First, we contacted key informants (i.e. community leaders) of the Vietnamese Men Who Have Sex with Men and Transgender Network and other civil business organizations (e.g. Vsmile company, iGirl group, Hai Dang club) to recruit participants for the FGDs. We asked them to search for potential candidates representing a diversity of demographic characteristics regarding age, sexual identity, education, and occupation. The leaders informed and asked members of their communities if they wanted to participate in the study, and then provided us with a list of possible participants who were willing to be contacted regarding participation in FGDs. These persons were invited by the research team, further informed about the study and asked if they were willing to participate. All study participants provided written informed consent.

### Focus group discussions

An FGD guide was developed for this study (Additional file 1) to address the research questions including attitudes towards the feasibility and uptake of HIV/STI interventions via online modalities (e.g. websites or smartphone applications, social network sites or emails); preferences and concerns regarding an online HIV/STI intervention. Social marketing principles such as audience analysis, channel analysis and product development were applied in developing the FGD questions [48]. Participants were divided into five FGDs sessions, that lasted between 90 and 120 min, with six to eight MSM in each session. We scheduled one FGD for young (18–22 years old) MSM (FGD1), one FGD for MSM living with HIV (FGD5) and three FGDs for all others (FGD 2–4). We continued data collection until data saturation was achieved. Adequate thematic saturation was determined when participants discussed consistently across the first three FGDs.

FGDs were held in a private room at the Hanoi Medical University to ensure the confidentiality and comfortability of participants. The FGDs were carried out in Vietnamese by LHN (a male Vietnamese public health practitioner) and HLTN (a female Vietnamese public health practitioner). Both facilitators are trained in qualitative study methodology and in conducting FGDs and have experiences from working with public health issues among MSM, youths, and persons living with HIV. First, LHN introduced briefly the purposes of this study and underlined the principles of FGDs to the participants, including mutual respect, privacy protection, that it was voluntary to share information and the option to withdraw at any point without consequences. After receiving written informed consents from participants, LHN led the FGDs and HLTN took participant observation notes and recorded the interview. The language used by the researcher was welcoming and gay-friendly to create a comfortable safe atmosphere. During FGDs, the participants freely expressed their thoughts and shared their experiences. Finally, they were asked to provide their information about socio-economic, HIV/STIs testing uptake, and HIV/STIs status using a short question sheet. Participants could at any time choose to end their participation without any consequences. Participants were served coffee and mineral water and given compensation for travel of a value of US$ 5.

### Analysis

The content of the FGDs was audio-recorded, transcribed verbatim, and translated into English by two members of the research team (LHN & LHTN). Directed content analysis was used to determine themes [49]. This approach started with the pre-identified questions that were utilized to develop the guideline of FDGs [49]. Two researchers (LHN, LHTN) initially read the FGDs thoroughly and then coded the transcripts keeping the preconceived research questions in mind, as well as identifying emerging themes. After coding each transcript, they were organized into three themes: 1) Experiences of current online resources for sexual health and HIV/STIs testing information; 2) Preferences for contents and features of eHealth interventions; and 3) Concerns for eHealth interventions use. There was high consistency in coding between the two researchers (LHN & LHTN). Differences in coding were resolved by discussion with a third researcher, also with experience in qualitative research among MSM and eHealth interventions (SS). Coded quotes were then organized according to the themes and a selection of quotes to include in the manuscript was made by the research team.

### Ethical approval

The study protocol, including the participant consent procedure, was reviewed and approved by the Institutional Review Board of Hanoi Medical University (Number 0618/HDDDDHYHN). Participants were not required to state any individual identifying information and outmost measures were taken to protect participants’ confidentiality.

## Results

### Sociodemographic characteristics of participants and experiences of HIV/STI-testing

**Fig. 1:**
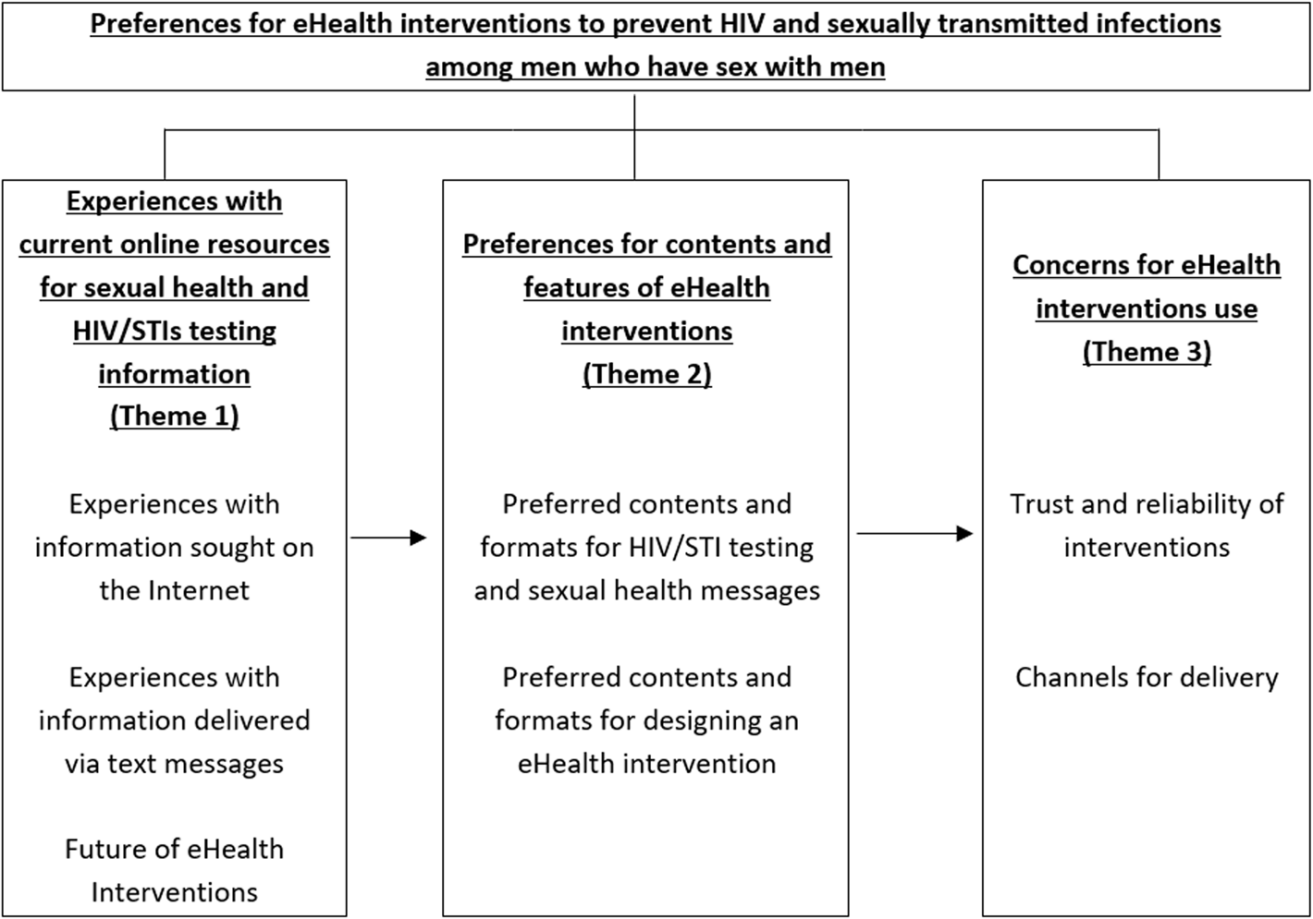
Code mapping into three main themes

Table 1 shows the demographic characteristics of the participants. A total of 35 MSM, aged between 18 and 40 years old and living in Hanoi, Vietnam, participated in five FGDs. Most participants attained at least high school education (91.4%) and were employed during the study (80%). Among the participants, five had never tested for STIs and four had never tested for HIV. All of the participants owned smartphones and personal computers. The emerging themes were organized as displayed in Fig. 1 and presented here.

**Table 1:** Demographic characteristics of participants

Demographic characteristics		n	%
Age group (years)	18–22	12	34.3
	23–30	18	51.4
	> 30	5	14.3
Education	< High school	3	8.6
	High school	14	40.0
	> High school	18	51.4
Occupation	Students	7	20.0
	Freelancer	7	20.0
	Business	6	17.1
	White-collar	9	25.7
	Artist	4	11.4
	Other	2	5.7
Marital status	Single	25	71.4
	Living with spouse/partner (female)	1	2.9
	Living with spouse/partner (male)	9	25.7
Peer-educator^a^	Yes	10	28.6
	No	25	71.4
Ever had a STI test	Yes	30	85.7
	No	5	14.3
	Unknown	0	0.0
Experience of STIs	No	22	62.9
	Syphilis	3	8.6
	Gonorrhea	2	5.7
	Chlamydia	2	5.7
	Trichomoniasis	0	0.0
	Hepatitis B	0	0.0
	Virus Herpes	4	11.4
	Human papillomavirus	2	5.7
	Do not remember	2	5.7
	Do not want to answer	1	2.9
Ever had a HIV test	Yes	31	88.6
	No	4	11.4
HIV status	Negative	26	74.3
	Positive	5	14.3
	Unknown	4	11.4

^a^ *Peer-educator: members in the MSM community who support health behavioral changes among their peers*

### Theme 1: experiences of current online resources for sexual health and HIV/STIs testing information

This section explores the experiences or habits of MSM in using eHealth approaches such as accessing the Internet or utilizing mobile devices to seek sexual health and HIV/STIs testing information.

#### Experiences with sexual health and HIV/STIs information sought on the internet

Overall, it was a common practice among participants in using eHealth tools such as personal computers or mobile phones to seek sexual health and HIV/STIs testing information on the Internet. Five men expressed that they preferred to use personal computers or laptops to find sexual health information, which could enable them to obtain a larger volume of information and more functions than on mobile phones*.* Especially, all of them were 30 years old or above. They stated that their jobs mainly worked with computers so they would want to use this device to find the information given its familiarity. However, in general, the vast majority reported that they frequently used smartphones as the primary tool for seeking HIV/STIs testing and sexual health information in Vietnamese on the Internet, even they owned personal computers or laptops. Mobile phones enabled them to go online irrespective of their location and update information or notifications immediately. Even one person who actively searched for information using laptops also expressed his preference in receiving information via a mobile phone.

For looking up sexual health information, participants used search engines (e.g. Google) and online social networks (e.g. Facebook (FB)) as well as “official” websites of MSM-related organizations or clinics as their primary sources. Social network sites were the most common given that the majority of participants stated their preference for these sources. One participant expressed that he believed in the information shared in the private FB group rather than other sources. This participant described his confusion when information found from other sources had different points of view, and he decided to be advised by seniors in his FB group. Problems regarding trustworthiness and quality of different online sources were also shared among other participants, especially in young participants, as they perceived them as “*…hard to believe*” or “*…difficult to follow or understand*”. These issues seemed not to be barriers when seeking information about HIV/STIs testing. Most participants could find practical information such as the symptoms of HIV/STIs, the address of the clinics, the cost of the tests, the organizations behind the clinics, and the procedures on the clinic websites. Moreover, participants could find contact information to medical experts in the clinics; but only three of them ever communicated with the medical providers through emails, telephones or web pages. In addition, the majority of participants expressed that online resources provided an environment that enabled MSM to share their experience about the quality of services, which could support them to make decisions around testing. Some participants expressed that they found information about HIV/STIs testing services on FB private groups. Furthermore, on the FB groups, they could find information about other types of testing such as HIV self-testing kits, which the participants “…*could not find in any clinic websites*”.

#### Experiences with sexual health and HIV/STIs information delivered via text messages

Participants reported that they rarely received messages for HIV/STI testing or sexual health. There was one participant reporting that he had received a short message services (SMS) message to remind him to take an HIV test when he participated in the research*.* Another person said that he received SMS messages after he had taken the HIV test at a clinic (e.g. the Hanoi Medical University MSM clinic) as a reminder to test again. He described the messages that had simple content including some texts to remind him of re-taking the test and the location of the clinic, and he expressed that he actually forgot the message. In general, most of them considered text messages as spams because the content was not attractive, and they were afraid of the lack of privacy or anonymity of the message. One participant expressed his doubt about the message:

*When someone send [text] messages to me, I will wonder why they send the message to me? Do they send to others? Posting on the web for publicity is more believable (A01, FGD1).*

#### Future of eHealth interventions

Overall, the majority of MSM in our FGDs agreed on the promising future of eHealth interventions among MSM in Vietnam because they believed that most MSM could access the Internet and mobile phones or smartphones easily. Two participants also reported that eHealth interventions could be implemented in the MSM population regardless of age group because they were now “…*willing to share”.* However, one participant concerned about the potential underutilization of this kind of online intervention and preferred face-to-face interventions or at least eHealth interventions with direct contact with medical experts.

*I think it's a bit difficult [to implement] because there is a vast amount of information on the Internet nowadays, so people do not concern that much. I think I need something that is not online, perhaps talk face-to-face or receive advice on something. Something should be provided as a phone number [of medical experts], so when people want to ask questions, they can call. (A03, FGD5).*

Despite expressing this preference, this participant still thought that eHealth interventions would be essential in the future because of its benefits. The majority of participants also expressed that they were willing to participate in the eHealth interventions if they were invited.

### Theme 2: preferences for contents and features of eHealth interventions

#### Preferred contents and formats for HIV/STI testing and sexual health messages

As aforementioned, some MSM reported that there was a huge amount of information with different quality on the Internet. When discussing their preferences for an HIV/STI testing and sexual health messages, several participants highlighted that the messages should be short, concise, accurate and straightforward: “*The [text] message needs to be short and concise so people do not need to spend a lot of time on it*” (A06, FGD5). For messages about reminding HIV/STIs testing, one participant described a procedure that he had experienced previously, and he was satisfied with:

*I like the system in the clinic at Hanoi Medical University. When they finish the examination, they take the phone, they call [me] directly and secretly inform about the appointment. (A03, FGD1)*

Participants expressed that the text messages about HIV/STIs testing should contain some information on logistics such as place of clinics, and time, procedures, and prices of the test, so that “*…persons receiving the message will have no questions left to ask”.* Moreover, participants had some specific suggestions of content and language for potential eHealth interventions. Furthermore, the content might include some keywords to describe the quality of the service such as “free”, “secure”, “fast”, or “accurate”. Participants expressed concerns that the content of text messages or SMS messages should not directly use perceived sensitive terms such as “HIV” or “STIs” in order to avoid that this could cause inconveniences if other people accidentally see the messages. This issue was considered critical as some participants reported that they did not want to receive SMS messages because of fear that other people would read them: “… *I’m scared that people would get to know about these messages.”* Likewise, words relating to “sex”, or “MSM” were described as sensitive, particularly in SMS messages. The issue of sensitive words was raised when participants were asked about sexual health messages:

*I want to note, for example, that for those who are unfamiliar with the sexual health contents, the messages should be sent via Facebook. Because only they know their FB account, they do not worry about that these messages are seen by other people. However, for people who are familiar with these contents, or for the messages sent via short message service or smartphone applications, the contents need to be encrypted. (A05, FGD3)*

Some participants recommended that the information for sexual health messages should not be repetitive because they would consider repeated messages as spams according to their previous experience. They were mostly interested in new information in the field:

*Repetitive messages, no new information, like advertisement. In the past, I treated [these messages] as spam and I banned the [phone] number [that sent these messages] (A04, FGD4).*

#### Preferred contents and formats for designing an eHealth intervention

Preferred contents and formats, as well as participants’ quotes for eHealth interventions, are presented in Table 2. When asked about the preferred contents, the participants requested a range of topics, which comprised information about HIV/STIs and testing, hotline counseling, sexual relationships, and safe sex practice, controlling risk behaviors such as substance misuse violence, discrimination, resources for MSM and support groups/platforms.

**Table 2:** Requested contents and format for tailored eHealth interventions for MSM

eHealth contents and format	Participants Quotes	Recommendations
**Contents**		
HIV/STI information	(1) I think the most important information is HIV and STI, how to prevent it. That will educate people to prevent infections, like the symptoms, how to detect, how to cope with that stuff. (A02, FGD4)	HIV/STIs information: symptoms, screening, diagnosis, treatment, prevention
HIV/STI testing	(2) Information about the list of MSM- friendly clinics, with the location of the consultation or treatment, is very good. Additional details may be the introduction of clinics, then connected. There should be lines available to call the counseling. (A01, FGD1)	List of clinics providing HIV/STIs testing, location, supporting hotline, process, procedures, online booking
Hotline counseling	(3) It should be a consultant line with a highly specialized consultant who can help callers, so that when I search the information on the Internet if I have questions, I can call them. (A02, FGD3)	Counseling hotline, real-time online counseling
Sexuality and relationships and safe sex	(4) Should put the situation between Bot^a^ and Top^a^ and show the problem. Find out about situations and emotions on both sides, to create mutual impact, why Bot do this and Top do that? This will create curiosity for the reader. Take other measures to increase the ability to make decisions (A04, FGD2) (5) I think information should include communication with a partner or anti-discrimination. (A03, FGD3)	Using role play scenarios to increase decision-making capacities, how to improve sexuality and sexual relationships, how to use condoms, how to cope with violence or discrimination
Controlling the behaviors	(6) Controlling the behavior again. I find that people in the control of certain types like drinking alcohol or drug is difficult. (A03, FGD5)	Controlling substance use
Resource for MSM	(7) I think it’s a good idea to include stories, but selectively. Because this community has already sunk, stories about good people, good things, make general people see that the community is beautiful, and people are more interested (A03, FGD1)	Life stories, MSM-related events
Support group	(8) I think you can create a forum for everyone to share or private groups on the Facebook. (A06, FGD4)	Social networks
**Format**		
Mood and tone	(9) For young people, it is necessary to have more fun, colorful content, while older people have to be mature. In general, the style should be neutralized for both (A03, FGD4).	Fun, colorful, happy, casual, straightforward
Mode of delivery	(10) I think the combination of text and image is better, the image should be less than text. I think the picture is very important. The more unique the image, the more attention it draws. (A01, FGD1)	Combine text, image, video clips, and infographic. Statistics for fear appeals Call to action
Access mode	(11) A website is preferred; however, you can consider designing that can fit the smartphone screen, so I can access anywhere. (A06, FGD2)	A website is a preferable choice, that can have the layout optimized for mobile devices.
Engagement strategies	(12) It should be attractive, as the style for adding some handsome boy pictures is very attractive. The interface should be friendly because some websites I have visited before cause a lot of trouble (A02, FGD4) (13) You can mention… what they will receive, something like economic, travel support. (A02, FGD2)	Attractive titles, images, and contents Create forum Rewards, financial incentives

^a^ *Explanation: Bot: Bottom – indicates receptive partner; Top: indicates insertive partner*

For HIV/STIs testing service, some participants recommended that the clinics should provide functions as an e-ticket or online bookings, which would enable them to easily book in advance online to come for HIV/STIs testing. One important function that the majority of participants would appreciate is a counseling phone hotline or real-time online counseling such as an online chat room. Via these forums support from medical experts in real-time could be made available. Some participants preferred to have secured and private forums or platforms only available for MSM that could build social networks with other MSM to share experiences and knowledge.

Notably, some men referred to use “handsome guys” images, while others suggested having stunning titles. They also advised that the messages delivered via the pictures or infographics were the most important and should be straightforward. Other participants recommended adding some statistics or facts that could make the readers aware of the need for HIV/STI testing or providing the tools to self-evaluate their risk of HIV/STI infection.

### Theme 3: concerns for eHealth interventions use

In this study, participants were also asked to reflect their concerns in implementing eHealth interventions as well as the manner to promote the use of eHealth interventions among MSM. There were two major issues that raised their most concerns including trust and reliability of interventions and channels for delivering interventions.

#### Trust and reliability of interventions

Some participants emphasized the trustworthiness of the organizations or individuals who sent the text/SMS messages/email messages or provided an eHealth intervention. These messages sent by well-known experts or organizations would increase their trustworthiness.

*First, I think I need to look at whether their site [mentioned in the message] is reputable, the second is the contact with the clinic. If I have any questions, I can send them what I want to have an answer on (A05, FGD5).*

This issue seemed to be more important among MSM living with HIV/AIDS. One participant in this group expressed that because they experienced the HIV-related stigma in their community; hence, if they received the messages from organizations and places where they were not familiar with, they might be scared. They were afraid that their private information was disclosed and then more people would know their HIV status. Another participant stated that because he migrated from a rural setting to Hanoi, he did not know much about which information source he should believe in. Therefore, if he received a message from an unknown organization, he would immediately ignore this message.

#### Channels for delivery

Some participants recommended sending the intervention message via email so that they could “…*read it later if needed”*. However, they would also re-underline that the title and content of the email should not include sensitive words such as “HIV”, “STI”, “sex” as aforementioned. Forums or groups targeting gay men on online social networks such as Facebook or dating applications such as Jack’D (https://jackdapp.com/) or Blued (https://www.blued.com/en/index.html) were recommended by most of the participants as channels to provide eHealth interventions for MSM. However, participants noted that if the eHealth interventions were delivered via Facebook or other social forums, it should not be opened for everyone to see. Private forums or groups within online social networks were better venues for information on sexual health to make it comfortable and confidential for the participants to read:

*You can post [the messages] to gay group sites or Facebook pages. But I think that Facebook private groups are the most suitable because if putting on the public page, many people who are not in the MSM community will feel uncomfortable. (A01, FGD1)*

*I recommend [the intervention should be delivered via] the dating app like Jack'D or Blued. For example, they can have a banner about the intervention, so if people are interested, they will click. There are a lot of people using these applications, so it will be effective to implement the intervention. (A01, FGD3)*

## Discussion

The current qualitative study describes the perspectives and expectations of MSM in Vietnam, which can be used to enable the development of eHealth interventions for this population. Our findings illustrated that currently available online information about HIV/STIs did not meet the needs described by participants, particularly regarding credibility and practicality. For design and implementation of future eHealth interventions MSM’s preferences for content, delivery methods and privacy concerns should be taken into account, which are important to optimize the effectiveness of such interventions.

Our study highlighted participants’ perceptions of the diversity of quality and practicality of information across online sources. Specifically, our participants reported being overwhelmed by HIV/STIs information on the Internet and therefore used strategies to look for information from senior peers who they believed in or perceived “official” sources, including websites of prestigious and locally well-known organizations such as Hanoi Medical University clinic and the Center for Disease Prevention and Control (CDC). Our finding was consistent with a study in China, which has reported that MSM could feel overwhelmed about the amount of online HIV/STI information and find it hard to navigate [50]. This is particularly important for those acquiring HIV/AIDS given that they were afraid of being disclosed their private information, which might lead them to be stigmatized. Therefore, the credibility of the information should be prioritized when developing eHealth interventions, which, as per our participants’ recommendations, could build the trustworthiness of these interventions among MSM.

Regarding the contents of eHealth interventions to attract MSM, participants recommended that the intervention should be interactive and informative with various topics rather than focusing only on HIV/STIs. For example, the majority of the participants emphasized a preference for interactive online platforms with real-time counseling and direct support from respectable medical experts, who could provide reliable information and help them to overcome barriers promptly in HIV/STIs prevention and care. This is consistent with a study among young MSM in Thailand [51]. Moreover, participants preferred information about testing services (location, price, and procedure), and reviews from previous clients, which supported them to make the decision to test. This finding aligns with a previous study in China, which suggested that offering tools to locate HIV/STIs testing clinics, setting appointments, and notifying testing results online could facilitate HIV/STIs testing among MSM [50]. Topics that would also captivate MSM included ways to improve sexual health and well-being or prevent substance misuse, violence, and discrimination. Participants were also interested in tools to evaluate their own risk of HIV/STIs to inform their decisions around safe sex and testing.

EHealth interventions would benefit from entertaining features to captivate MSM’s attention, combining texts, clips, pictures, and infographics compatibly for website contents. Straightforward messages were preferred, however, there were caution around sensitive language around sex and HIV/STIs. These statements were somewhat contradicting between participants and it seems that there was diversity in preferences around language. However, the information in the messages or websites provided to them should be accurate, practical, short, concise and easy to understand, which have been seen in another study among Black MSM in the United States [47]. Therefore, careful piloting of eHealth interventions will be of importance to ensure comfortability with the content. It is of uttermost importance to avoid messages causing fear or discomfort [52, 53].

The diversity of channels to deliver messages and eHealth interventions are also important to consider. Indeed, with a variety of devices used, several Internet-based approaches might be considered to provide interventions such as websites, social network sites namely Facebook, or even SMS messages. Regarding messages, the majority of the participants showed interest in receiving information via private groups on Facebook, gay forums or dating applications (e.g. Jack’d or Blued which were mentioned as the most popular apps). A few participants preferred to receive messages via email because then they could save and read it later, other participants reported they did not use email frequently and therefore would not see such messages. Previous trials in China found that interventions delivered through email did not reach a sufficient efficacy to change the behaviors, which might be due to a lack of interactive activities and individualized messages for participants’ engagement [54, 55].

In terms of devices used, some participants preferred to access websites by using laptops or personal computers because they could process greater volumes of information on a larger screen, especially among MSM with high age. Since the prevalence of smartphone owners in MSM in Vietnam is high, particularly in urban areas (91.5% [46]), optimizing designs in different platforms (i.e. mobile phone-based and traditional web-based) should be taken into consideration when developing the intervention. A web design approach such as Responsive Web Design could be a potential strategy as a website can change its interface to be optimized according to the users’ devices [56]. Smartphone applications were also one preferred platform to enable flexible usage at any time. In literature, this approach has been supposed to encourage behavioral changes in a previous study among Black MSM in the United States [47]. Providing HIV/STIs information in these ways could offer the option for MSM to access either simple tools (e.g. seeking testing site locations) or more extensive information (e.g. about how to improve sexual relationships, dealing with violence or substance abuse, or HIV/STIs treatment) [47, 50, 51].

It should be noted that despite a wide acceptance in receiving eHealth interventions, MSM reported privacy concerns when accessing health information on the Internet and more so when receiving information through their mobile phones. Specifically, they worried that their friends, colleagues or families might accidentally look at their mobile phones or computers and see HIV/STI-related messages. The advantage of the Internet is to increase the accessibility of interventions among MSM in highly stigmatized settings [36, 37]. Thus, it is cautious to avoid any sensitive texts when delivering intervention messages. Possible solutions suggested to address these issues include using code-phrases or neutral contents, which could be complemented by links to further information that could be accessed in a private environment. This finding highlighted the importance of piloting sensitive messages carefully as well as the security features of the interventions to protect participants’ confidentiality [47, 50].

### Limitations and strengths

There are several limitations in this study. We recruited participants with a diversity of socioeconomic backgrounds, however, we might not have reached all types of MSM rather only openly gay men and a large group that tested themselves already. In addition, participants lived in Hanoi, a metropolitan in Vietnam. Significant differences in preferences might be observed in MSM living in rural areas, where the stigmatization towards homosexuality may be higher. It should be cautious when applying the results of this study in other settings. Moreover, the interventions which were developed based on the finding of this study should take into account the disparity in different settings of Vietnam. Lastly, the nature of a qualitative study limits the generalizability of the results. However, measures were taken to ensure the validity of the findings by creating a safe and friendly atmosphere in confidential environments to encourage participants to share their consensus as well as dissonance within FGDs. In addition, the participants actively discussed sensitive topics on sexual behavior and HIV/STI testing experiences, suggesting that they felt sufficiently comfortable to share their stories. Finally, the redundancy of contents in all FGDs indicates the saturation of information on this topic.

## Conclusion

This study indicates a high acceptance and perceived need for online HIV/STI intervention in the MSM population in Hanoi, Vietnam. Participants preferred online resources from well-known trusted organizations with easy to understand information about HIV/STIs, testing, and treatment using platforms that are available both through smartphones and personal computers. They preferred to access this information at a time and place of their own choice. Privacy concerns were mostly related to cases of sensitive information being sent in text messages to their phone. The findings might be useful to increase the efficacy and acceptance of eHealth interventions among MSM in Vietnam.

## Supplementary information

**Additional file 1.** Focus Group Discussion Guide. A questionnaire that were used for guiding the focus group discussion

## Data Availability

The data that support the findings of this study are available from the Hanoi Medical University but restrictions apply to the availability of these data, which were used under license for the current study, and so are not publicly available. Data are however available from the authors upon reasonable request and with permission of Hanoi Medical University.

## Abbreviations

AIDS: Acquired immunodeficiency syndrome
eHealth: Electronic health
FGD: Focus group discussion
HIV: Human immunodeficiency virus
MSM: Men who have sex with men
SMS: Short message service
STIs: Sexually transmitted infections
