# Supplementary material for: An exploratory assessment of the preference for eHealth interventions to prevent HIV and sexually transmitted infections among men who have sex with men in Hanoi, Vietnam
Source: BMC Public Health. 2020 Sep 11;20:1387. doi: 10.1186/s12889-020-09449-z (PMC7488431; doi:10.1186/s12889-020-09449-z)
Supplement: Supplementary file 1 — Additional file 1. Focus Group Discussion Guide. A questionnaire that were used for guiding the focus group discussion [file 12889_2020_9449_MOESM1_ESM.doc]

**MEN WHO HAVE SEX WITH MEN**

**TEMPLATE FOR CONDUCTING FOCUS GROUP DISCUSSION**

Research Staff ID:

Translator ID: Date:

Informant ID: Place of Interview:

Consent obtained? Y / N Consent version used:

Notes:

**Total Participant Time Required: 90 – 120 minutes**

Total number of participants: 7-10 people

**EQUIPMENT NEEDED:**

- Paper and pens for everyone
- An easel
- Markers
- Cards for names participants want to use
- Recording equipment
- A facilitator and assistant

| OVERALL QUESTIONS TO ANSWER IN FOCUS GROUP DISCUSSION: **Reminder to facilitator:**  The purpose of this focus group among men who have sex with men (MSM) is to determine the following:   - Identify the needs of online interventions for MSM population among stakeholders - Identify the most appropriate approach for designing online intervention for MSM population |
| --- |

**FOCUS GROUP: DISCUSSION GUIDE**

**Facilitator’s welcome, introduction and instructions to participants**

**Welcome** and thank you for volunteering to take part in this focus group. You have been asked to participate as your point of view is important. I realize you are busy and I appreciate your time.

**Introduction:** This focus group discussion is designed to assess your current thoughts and feelings about the needs of online interventions for MSM population, and the appropriate approach to design these interventions. The focus group discussion will take no more than two hours. May I tape the discussion to facilitate its recollection? (if yes, switch on the recorder)

**Anonymity:** Despite being taped, I would like to assure you that the discussion will be anonymous. The tapes will be kept safely in a locked facility until they are transcribed word for word, then they will be destroyed. The transcribed notes of the focus group will contain no information that would allow individual subjects to be linked to specific statements. You should try to answer and comment as accurately and truthfully as possible. I and the other focus group participants would appreciate it if you would refrain from discussing the comments of other group members outside the focus group. If there are any questions or discussions that you do not wish to answer or participate in, you do not have to do so; however please try to answer and be as involved as possible.

**Ground rules**

- Turn off you cell phones during the discussion.
- Please try to protect each other’s confidentiality (feel free to share what you heard here but don’t link it back to someone here specifically). Some of you may know each other. Even if you do, please respect each other’s privacy and confidentiality by not mentioning each other’s presence in the group to other people. Everyone participant in this group is living with HIV and it is critically important that people respect each other’s decisions to share or keep this information private from others. So, please do not share information about people’s HIV-status to anyone outside of this room.
- Please respect each other and each other’s opinions. There is no incorrect way to feel or think here and we want to encourage everyone to have the opportunity to share.
- Finally, please try to speak one at a time, so we can listen what other colleagues have to say. This will also make it easier to transcribe our discussions accurately.

**Warm up**

- - - - On the paper in front of you, please fill your demographic characteristics. [PAUSE]

**Guiding questions**

**[Experiences and Need of Online intervention]**

I would like to spend a few minutes talking about ways people use Internet to seek information about the way to protect their sexual health. We talked about different things people try to do. I would like to ask you all to share your opinions on the kinds of support that might help people to adopt some of these strategies into their sexual health approaches.

Q1. Which sources do you often use for finding information about sexual health and HIV/AIDS prevention?

⇒PROBE: observing the usage of smartphone/laptop, etc. among participants

Q2. What do you think if you receive cell phone messages or email messages or Zalo/Viber/Tinder/Grindr that act as a reminder or motivator for you to take care of your sexual health. Would you want those? How often? What would they look like?

⇒PROBE: Which factors should be cautious when sending the message or information?

Q3. How do you think about the implementation of HIV/STI prevention and control interventions for MSM population via online approaches such as Internet, web, smartphone?

**[Online intervention design]**

Q4. Which is the best way to approach MSM population?

⇒PROBE: Do you have any suggestions to reach MSM population as much as possible?

Q5. Which information should be provided in this intervention?

Q6. Which modality should be used to provide this information?

Q7. Which channel can be used to provide this information to MSM population?

**Concluding question**

Q8. What recommendations do you have for developing an online intervention program that tries to help MSM to facilitate safe sex and HIV/STI testing that have not yet been discussed? Is there anything you might want to add?

**Conclusion**

- Thank you for participating. This has been a very successful discussion
- Your opinions will be a valuable asset to the study
- We hope you have found the discussion interesting
- If there is anything you are unhappy with or wish to complain about, please contact the PI or speak to me later
- I would like to remind you that any comments featuring in this report will be anonymous
- Before you leave, please hand in your completed personal details questionnaire
